# Methods for the frugal labeler: Multi-class semantic segmentation on heterogeneous labels

**DOI:** 10.1371/journal.pone.0263656

**Published:** 2022-02-08

**Authors:** Mark Schutera, Luca Rettenberger, Christian Pylatiuk, Markus Reischl

**Affiliations:** Institute for Automation and Applied Informatics, Karlsruhe Institute of Technology, Karlsruhe, Baden-Württemberg, Germany; Vellore Institute of Technology: VIT University, INDIA

## Abstract

Deep learning increasingly accelerates biomedical research, deploying neural networks for multiple tasks, such as image classification, object detection, and semantic segmentation. However, neural networks are commonly trained supervised on large-scale, labeled datasets. These prerequisites raise issues in biomedical image recognition, as datasets are generally small-scale, challenging to obtain, expensive to label, and frequently heterogeneously labeled. Furthermore, heterogeneous labels are a challenge for supervised methods. If not all classes are labeled for an individual sample, supervised deep learning approaches can only learn on a subset of the dataset with common labels for each individual sample; consequently, biomedical image recognition engineers need to be frugal concerning their label and ground truth requirements. This paper discusses the effects of frugal labeling and proposes to train neural networks for multi-class semantic segmentation on heterogeneously labeled data based on a novel objective function. The objective function combines a class asymmetric loss with the Dice loss. The approach is demonstrated for training on the sparse ground truth of a heterogeneous labeled dataset, training within a transfer learning setting, and the use-case of merging multiple heterogeneously labeled datasets. For this purpose, a biomedical small-scale, multi-class semantic segmentation dataset is utilized. The heartSeg dataset is based on the medaka fish’s position as a cardiac model system. Automating image recognition and semantic segmentation enables high-throughput experiments and is essential for biomedical research. Our approach and analysis show competitive results in supervised training regimes and encourage frugal labeling within biomedical image recognition.

## 1 Introduction

Today, biomedical image recognition and semantic segmentation are successfully driven by deep learning approaches and supervised training of neural networks [1]. Learning feature representations from data enables the identification and quantification of patterns in biomedical data [2]. Biomedical applications and especially studies rely on high-performing semantic segmentation neural networks [3, 4]. A variety of neural network architectures have addressed semantic segmentation. In biomedical image segmentation, deep symmetric convolutional neural networks, such as V-net [5], and U-Net [6] dominate the algorithmic state-of-the-art. In supervised deep learning approaches, these architectures are driven by large-scale, homogeneous labeled datasets [7–9]. Homogeneity means that the ground truth for each sample of the dataset includes a label for each of the specified classes. Heterogeneity defines a ground truth in which different samples might miss labels for a present class or include labels for specific classes only. Supervised learning methods from the outset are subject to restrictions when applied to heterogeneous labeled data. Heterogeneous labeled data as defined by [10] is to be understood as partially labeled data. As a result, supervised training on heterogeneous data means reducing the number of available data samples for training, limiting the task’s scope to a reduced set of classes, or conclusively an unfeasible training.

Biomedical image datasets for semantic segmentation are commonly small-scale, including heterogeneous, pixel-wise labels. These heterogeneous datasets are regularly discarded during data preprocessing and cleaning before supervised training. It is tedious to transfer a heterogeneous dataset into a homogeneous dataset by supplementing labels. Usually, such datasets are waived before training. Nevertheless, these biomedical datasets carry domain knowledge that asks for modeling, is challenging to obtain, and expensive to label, and their applications range from image classification to semantic segmentation. Therefore, different approaches have been taken in the related work to cultivate heterogeneous datasets for supervised neural network training. One field considers multi-modal learning where the input samples are from different source modalities such as audio, image, text, and video. Another field focuses on multi-task learning, where the neural network has to perform multiple different tasks. Most commonly, neural networks in this field are based on learning a joint representation, or a shared feature space combined with either separate encoding paths in the multi-modal setting [11], or separate classification output layers in the multi-task learning [12–14]. Consequently, much ground truth information is lost if the data is only partially labeled, which is the case with heterogeneous data.

The most basic solution to deal with missing labels is to drop the loss of unknown entities in the objective function, which merely overcomes the technical limitation of a loss value not being determinable if the ground truth is missing [15]. Marginally more advanced is to treat missing class labels as background, which, however, introduces the problem that it is assumed that everything unlabeled must be background, which is most often not the case [16–18]. A more sophisticated approach is to train a shared feature space across different datasets or classes while introducing fine-tuned sub-classifier heads, which enabled the merging of multiple datasets. Still, this approach treats unknown labels as background without leveraging any additional knowledge [19]. More data-focused approaches try to incorporate knowledge by using the similarity between classes to merge them, which requires the precondition that multiple classes are similar in their appearance. Further, merging multiple classes leads to training a more general (and less complex) task [20]. Finally, a promising yet straightforward approach is to take full advantage of the fact that virtually all semantic segmentation tasks are multi-class problems. All data points are assigned a single, unambiguous class. This realization leads to an objective function that utilizes the mutually exclusive nature of ground truth masks in semantic segmentation [10, 21].

All approaches mentioned above try to cope with unlabeled information in datasets by altering established methods or exploiting supervision cues in the data. This work combines both methods within the training process by utilizing explicit annotation information and, in particular, the information implicitly available in a sample.

Wasting heterogeneous labeled data for biomedical applications should not remain the best practice. Instead, this work proposes an approach for the frugal labeler and biomedical image recognition engineer to train neural networks for multi-class semantic segmentation (with U-Net) on heterogeneously labeled datasets. The novel combined objective function is presented, which represents a modified version of a sample dropping objective function [16, 17], extended by the class-asymmetric (CA) objective function [10], to train on heterogeneous labeled datasets. The approach is implemented to train on heterogeneous labeled data according to training settings common in biomedical semantic segmentation: training on a heterogeneous labeled dataset, transfer learning for domain adaptation by introducing an additional class and training on multiple datasets with heterogeneous labels. At the same time, our results prove that our method’s performance matches supervised training on a homogeneous labeled dataset. In detail, this work’s contributions are:

- A biomedical homogeneous labeled benchmark dataset covering the central vascular system (heart, atrium, bulbus) in Medaka is provided to enable reproducibility of the experiments.
- A software package for the synthetic generation of heterogeneous datasets is provided; this allows for the homogeneous dataset’s configurable adaptation.
- A segmentation performance baseline is created by supervised multi-class semantic segmentation with U-Net on the homogeneous labeled dataset of the central vascular system of the Medaka.
- A novel objective function is developed and tested on three different dataset characteristics.

*With these contributions, we enable the practitioner and the research community to reduce label efforts for multi-class segmentation by making heterogeneously labeled data deployable for supervised training. We aim to create awareness for the value and possibility of deploying heterogeneously labeled datasets in biomedical applications and demonstrate how to do so. All code and data employed in this paper are open-sourced*.

## 2 Methods and data

### 2.1 Overview

First, the approach is presented, which transforms a homogeneous dataset, where every instance is labeled with all classes into a heterogeneous dataset, where samples may only be partially labeled. Second, a novel combined objective function is introduced, which is tailored to operate on heterogeneously labeled datasets. For this a dataset $\left(X,\tilde{Y}\right)$ is employed, where each sample label pair $\left(x,\tilde{y}\right)\in \left(X,\tilde{Y}\right)$ consists of a sample **x**, which is an RGB image with the dimensions (*w*, *h*, 3) and a ground truth label $\tilde{y}$ with dimensions (*w*, *h*, *C*), where *w* is the width and *h* the height of the image sample and its corresponding ground truth label and *C* is the number of classes. The number of sample label pairs in the dataset is defined as *S*. Subsequently, we introduce our biomedical dataset of the central vascular system of the Medaka fish, which serves as a benchmark for the conducted experiments.

### 2.2 Heterogeneous label dataset generation

There is a need to generate multiple distinct datasets with varying degrees of missing labels based on a common source for structured label ablation experiments on a heterogeneous dataset. While it would be possible to conduct experiments on multiple datasets that are inherently heterogeneous and stem from multiple domains, such approaches are not feasible if we want to draw conclusions on the deployed methods and the influence of missing labels. When drawing samples from the same source dataset, the presented methods will be decisive in evaluating different hyperparameter configurations in the conducted experiments.

To obtain a heterogeneous dataset from a predefined homogeneous source $\left(X,\tilde{Y}\right)$, the class labels, which should potentially be dropped, need to be defined as a subset $d=\{d_{i}\in N\;|\;d_{i}\leq C\}$ of the number of all classes. For each class label in **d**, a defined absolute share of labels P∈N with P≤S is dropped from the source dataset labels. This means that for every *d*_*i*_ ∈ **d**, P randomly chosen labels are removed from the ground truth labels $\tilde{Y}$. The relative share of removed labels is described by ρ=P/S. With this procedure, we can obtain a large amount of unique, synthetically produced heterogeneous datasets which all stem from the same data source. For implementation details see Chapter 4 and https://github.com/Heterogeneous-Semantic-Segmentation/Utilities-for-The-Frugal-Labeler.

### 2.3 Biomedical image segmentation with U-Net

The U-Net [6] is a deep, symmetric fully convolutional neural network, which follows the typical architecture of encoder-decoder networks. The encoder produces feature maps by combining convolutional and pooling operations while the decoder employs transposed convolutions to densify the feature maps and generate segmentation masks. Additionally, features at multiple resolutions of the encoder path are used in the decoder to enhance the generated segmentation masks. The architecture was initially motivated by semantic segmentation tasks on small-scale biomedical image datasets. This work builds upon previous heart segmentation experiments with the U-Net [3]. The architecture is extended to expect an RGB image, not a grayscale image, as an input and to classify multiple classes instead of just one. The input is expected to be a tensor of the shape (*w*, *h*, 3), and the output is a tensor of the shape (*w*, *h*, *C*). Each coordinate (*u*, *v*) with *u* ∈ {0, …, *w*} and *v* ∈ {0, …, *h*} in the output tensor contains a normalized probability distribution of all classes (softmax). The model and training pipeline are publicly available (see https://osf.io/uyk79/).

### 2.4 Objective function

The objective function used in this work is tailored to enable learning on heterogeneous labels and stabilize the learning process by exploiting implicit information of unlabeled classes. The channels, meaning class labels, and the normalized predicted output of overall classes for each pixel are utilized when calculating the objective function’s output.

In the following ${\tilde{Y}}_{w\times h\times C}$ is defined to be the ground truth tensor and ${\hat{Y}}_{w\times h\times C}$ the prediction of the U-Net. Facing heterogeneously labeled data combined with the fact that the U-Net expects an input tensor with constant size requires marking missing labels in another way than simply dropping them. For that matter, a binary label mask vector $m\in R^{c}$, with *c* = {1, …, *C*} is introduced, which indicates whether a ground truth mask exists for any of the *C* classes. If a ground truth mask exists, *m*_*c*_ is 1 and 0 if not.

#### 2.4.1 Channel optimization

The proposed objective function is a composition of two individual objective functions. The first component of the objective function is optimizing each channel of the prediction tensor $\hat{Y}$ to be closer to the ground truth tensor $\tilde{Y}$ with each step, therefore horizontal optimization. For this, the Dice-Sørensen Coefficient (*DSC*) is employed. The *DSC* divides the area of overlap between the segments of two segmentation masks by the overall number of pixels in both masks (see Eq 1)

$$\begin{array}{c} DSC\left(c,\tilde{y},\hat{y}\right)=\frac{2\sum_{u=0}^{w}\sum_{v=0}^{h}\left({\tilde{y}}_{u,v,c}\cdot {\hat{y}}_{u,v,c}\right)+\epsilon }{\sum_{u=0}^{w}\sum_{v=0}^{h}{\tilde{y}}_{u,v,c}+\sum_{u=0}^{w}\sum_{v=0}^{h}{\hat{y}}_{u,v,c}+\epsilon }. \end{array}$$ (1)

A small positive number *ϵ* = 10^−7^ is added to the numerator and denominator in the calculation of *DSC* to avoid division by zero. The *DSC* statistic is embedded into a modified Dice loss $L_{DSC}$ (see Eq 2). The quantity (1 − *DSC*) is summed over all classes in $L_{DSC}$. For every mask the value of (1 − *DSC*) is multiplied by *m*_*c*_, which means that the current value is only taken into account if the specific mask exists (*m*_*c*_ = 1). After that the accumulated loss is normalized with respect to the number of present masks, so $L_{DSC}$ is always scaled according to how many masks are present

$$\begin{array}{c} L_{DSC}\left(\tilde{y},\hat{y}\right)=\frac{\sum_{c=1}^{C}m_{c}\;\left(1-DSC\left(c,\tilde{y},\hat{y}\right)\right)}{\sum_{c=1}^{C}m_{c}}. \end{array}$$ (2)

#### 2.4.2 Probability distribution optimization

The second component of the combined objective function assumes that pixels do not belong to two classes simultaneously in a multi-class segmentation problem. For this, the class asymmetric loss (*CAL*) is employed. This function only observes masks that do not have a ground truth value. If a pixel within a mask has a high confidence value, but it is known that it belongs to a different, labeled class, the function will deliver a high value. So, if a ground truth value is defined for a pixel, it is known that the pixel cannot belong to an unlabeled mask. However, if the pixel has no ground truth defined, it is not possible to determine whether the pixel belongs to the unlabeled mask. To calculate *CAL*, the values of the given mask index *c* are summed over all coordinates (see Eq 3). For each coordinate (*u*, *v*), the pixel at the same position in all mask indices *z*, except for the current mask *c*, is observed and the predicted value ${\hat{y}}_{u,v,z}$ at that coordinate is observed. An activation function *f*(.) (such as sigmoid) transforms the prediction to constrain the range of possible output values. Afterward, the value is multiplied by (1 − *m*_*z*_) only to take those masks into account, in which the ground truth is not set. Finally, the summation is multiplied by the ground truth value ${\tilde{y}}_{u,v,c}$. Since ${\tilde{y}}_{u,v,c}$ is 1 if the mask index *c* is the correct class for the pixel at position (*u*, *v*), and 0 otherwise, this multiplication ensures that the summation is only taken into consideration if ${\tilde{y}}_{u,v,c}$ is the correct class for this pixel

$$\begin{array}{c} CAL\left(c,\tilde{y},\hat{y}\right)=\sum_{u=0}^{w}\sum_{v=0}^{h}({\tilde{y}}_{u,v,c}\cdot \sum_{z\in \{1,\ldots ,C\}\{c\}}f\left({\hat{y}}_{u,v,z}\right)\cdot \left(1-m_{z}\right)). \end{array}$$ (3)

*CAL* is then used in $L_{CAL}$ (see Eq 4) to calculate the second component of the composed objective function. As in $L_{DSC}$, $L_{CAL}$ is determined by calculating the sum over all classes and only taking into account the classes for which a mask exists (*m*_*c*_ = 1)

$$\begin{array}{c} L_{CAL}\left(\tilde{y},\hat{y}\right)=\sum_{c=0}^{C}m_{c}\;CAL\left(c,\tilde{y},\hat{y}\right). \end{array}$$ (4)

If the background class is determined as the class in which none of the *C* defined classes is present, it needs to be excluded in the calculation of $L_{CAL}$. This is necessary since this naive definition of the background class makes it impossible to distinguish between unlabeled pixels and actual background.

#### 2.4.3 Combined objective function

The combined objective function is designed as:

$$\begin{array}{c} L=\left(1-\alpha \right)\;L_{DSC}+\alpha L_{CAL}, \end{array}$$ (5)

With *α* ∈ [0, 1), is the weight between the two individual loss functions. The weighting factor *α* adjusts the influence between the horizontal, channel-wise optimization of $L_{DSC}$ and vertical, probability distribution-wise, optimization of $L_{CAL}$. The value *α* = 1.0 is not considered to be valid since $L_{CAL}$ only makes statements about unlabeled data and hence would not be able to operate independently.

**Fig 1:**
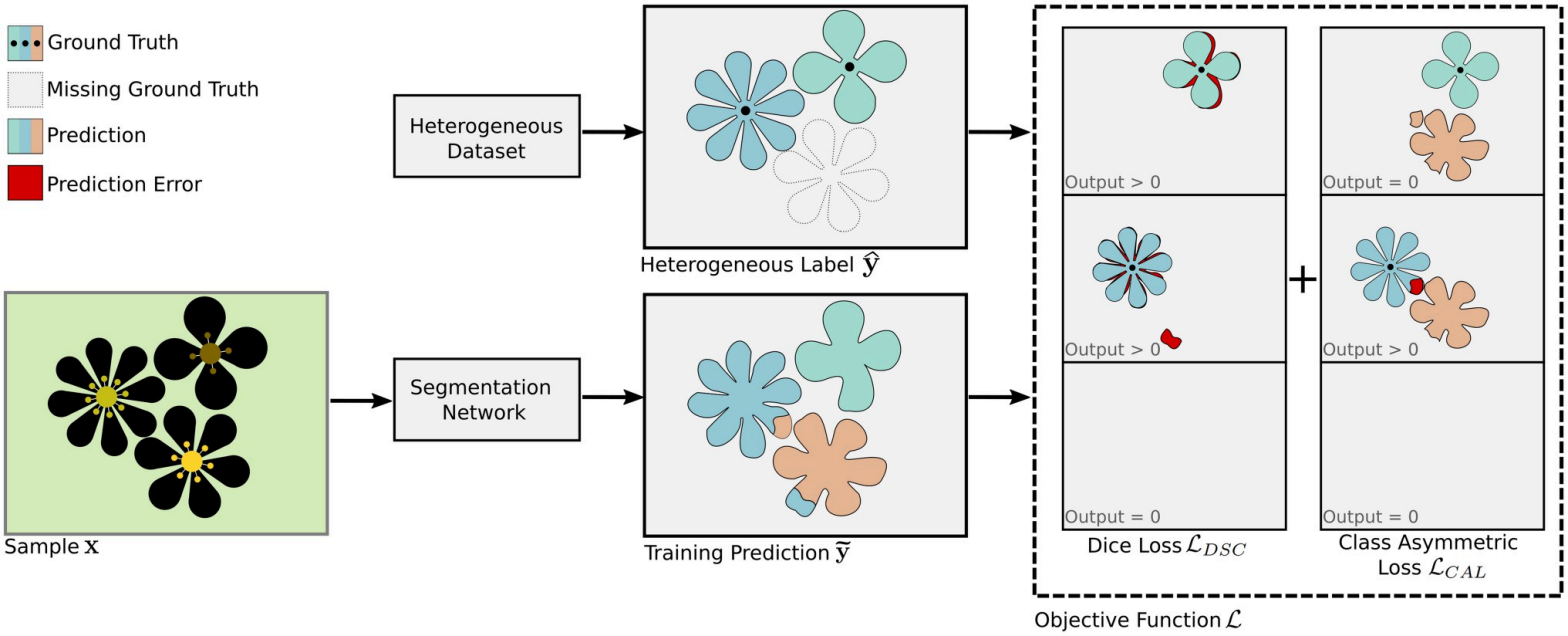
Visualization of the combined objective function. An exemplary sample **x** is fed into the network, which outputs a training prediction $\hat{y}$. Each class is denoted with a unique color. The corresponding ground truth label $\tilde{y}$ is missing the class on the lower right. The dotted line marks where the label would be in the sample. The dashed box on the right denotes L, with the output for both objective functions $L_{CAL}$ and $L_{DSC}$. Prediction errors are marked as red areas. The number in the lower-left corners indicates the approximate output for the respective objective function. Since there is a prediction error in both given classes, the Dice loss will output a value L>0 for them and 0 for the unlabeled class since it cannot make a statement without the ground truth mask. The class asymmetric loss will only output a value L>0 if an unlabeled class’s predicted segmentation mask intersects with a given class’s ground truth mask. Since the network predicted a small portion of the unlabeled class as part of the class in the center-left, the output will be L>0 for this class. For the other labeled class, no intersection occurred, and hence the output is 0. Since the unlabeled mask does not contain a ground truth, it is not evaluated by the class asymmetric loss, which will result in an output of 0.

The combined objective function L is tailored to take advantage of heterogeneously labeled datasets, with two individual loss functions which complement one another, each focusing on optimizing one dimension (horizontal or vertical) of the output tensor. In Fig 1 the calculation of L is depicted.

### 2.5 Performance frugality ratio (*PFR*)

A novel metric is introduced to measure the similarity between ground truth labels and predictions, under consideration of the degree of heterogeneity in the given data and an existing statistic M (such as *DSC* or the Intersection over Union (*IoU*)). The Performance Frugality Ratio (*PFR*) divides the given statistic by the number of available labels n=S-P, which is the difference between the number of overall sample-label pairs *S* and the share of dropped labels P (see Eq 6)

$$\begin{array}{c} PFR\left(M\right)=\frac{M}{n}. \end{array}$$ (6)

Since *PFR* takes heterogeneity in the dataset into consideration, the calculation is more profound than the used statistic alone. More precisely, if fewer samples need to be labeled to achieve the same performance for a given statistic, the same result is achieved with less information which means better performance.

### 2.6 Extended heartSeg dataset

The dataset **X** of this work is an extension of the heartSeg dataset [3]. Each sample **x** ∈ **X** is an RGB image capturing the heart region of Medaka (Oryzias latipes) hatchlings from a constant ventral view. Since the body of Medaka is see-through, noninvasive studies regarding the internal organs and the whole circulatory system are practicable [22]. A Medaka’s heart contains three parts: the atrium, the ventricle, and the bulbus. The atrium receives deoxygenated blood from the circulatory system and delivers it to the ventricle, which forwards it into the bulbus. The bulbus is the heart’s exit chamber and provides the gill arches with a constant blood flow. The blood flow through these three chambers was captured in 63 short recordings (around 11 seconds with 24 frames per second each) in total, from which the single image samples **x** ∈ **X** are extracted. The dataset is split into training and test data following the heartSeg dataset [3] with *n*_*train*_ = 565 samples in the training set **X**_*train*_ and *n*_*test*_ = 165 samples in the test set **X**_*test*_. The RGB image samples have a 640 × 480 pixels resolution.

#### 2.6.1 Data labeling

Each **x** ∈ **X** in the heartSeg dataset possesses an associated ground truth mask $\tilde{y}\in \tilde{Y}$. Initially, $\tilde{Y}$ solely contains the ventricle class **V**. Within this work, we extended the labels by two semantic classes: the bulbus **B** and the atrium **A** of the medaka hatchlings’ circulatory system.

**Fig 2:**
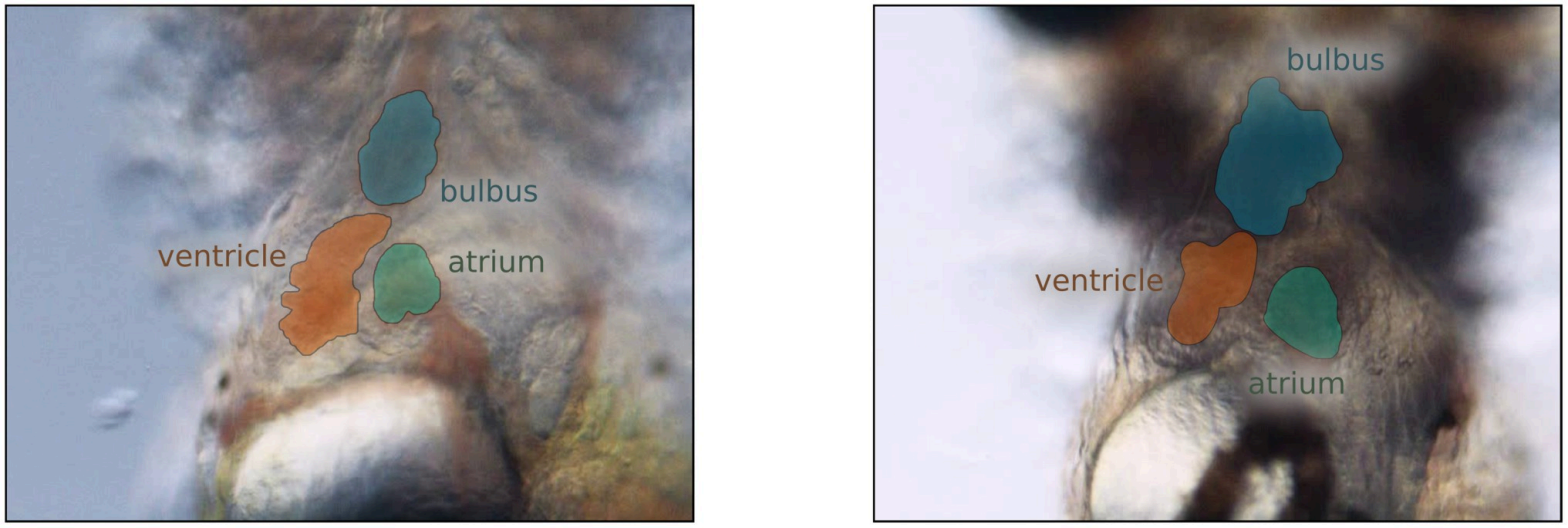
Labels of the extended heartSeg dataset. Displaying image masks for the three semantic classes overlaid on two image samples of the medaka hatchlings’ cardiac system as part of the extended heartSeg dataset. The orange label masks depict the ventricle **V** (based on the heartSeg dataset [3]). The blue and green label masks depict the respective, newly added semantic classes bulbus **B** and atrium **A**.

Prior to our additional labeling, each $\tilde{y}\in \tilde{Y}$ is a binary matrix with the same dimensions as its associated sample where each pixel ${\tilde{y}}_{u,v}$ indicates whether it belongs to the ventricle class **V** (${\tilde{y}}_{u,v}=1$) or the background (${\tilde{y}}_{u,v}=0$). After labeling, each $\tilde{y}$ contains the label masks (*V*,*B*,*A*) for all three classes (see Fig 2). The mask for each class is an individual binary image mask, therefore the extended dataset contains a total of (*n*_*train*_+ *n*_*test*_)*3 = 2, 190 label masks. It takes around 25 seconds to generate a single label mask for an individual class. The overall time spent to extend the heartSeg dataset by the atrium and the bulbus class was around 10 hours. For scale, extending this dataset to *n*_*c*_ = 5 classes and approximately *n*_*s*_ = 5, 000 samples would result in *n*_s_*n_*c*_ = 25, 000 label masks and a total labeling time of around 174 hours for all samples.

Even though Medaka is nearly transparent, the heart chambers are only clearly identifiable in the recordings if filled with enough blood. The cardiac cycle makes it difficult to reliably label a chamber if it is only partially filled with blood and very challenging if it contains little blood (end-systolic). The extended heartSeg dataset is available for download here (https://osf.io/uyk79).

## 3 Results

Four experiments evaluate how the approaches for training on heterogeneous labels influence the multi-class semantic segmentation performance. The first experiment (see Subsection 3.1) is a general ablation study in which labels from all classes are dropped. The second experiment (see Subsection 3.2) simulates a situation in which a dataset labeled with one class is extended by a second class. The extension formulates a transfer learning task [23] since the knowledge available by the labels of the first class is used to transfer the knowledge and learn on the second class. The third experiment (see Subsection 3.3) is a variation of the second experiment, in which each sample is only associated with a single label mask. It thus only holds information on one of the two classes simultaneously. The fourth experiment (see Subsection 3.4) investigates the contribution of the two individual loss functions, which form the combined objective function, by modifying the weight between them.

### 3.1 Label-effort reduction in multi-class semantic segmentation

**Fig 3:**
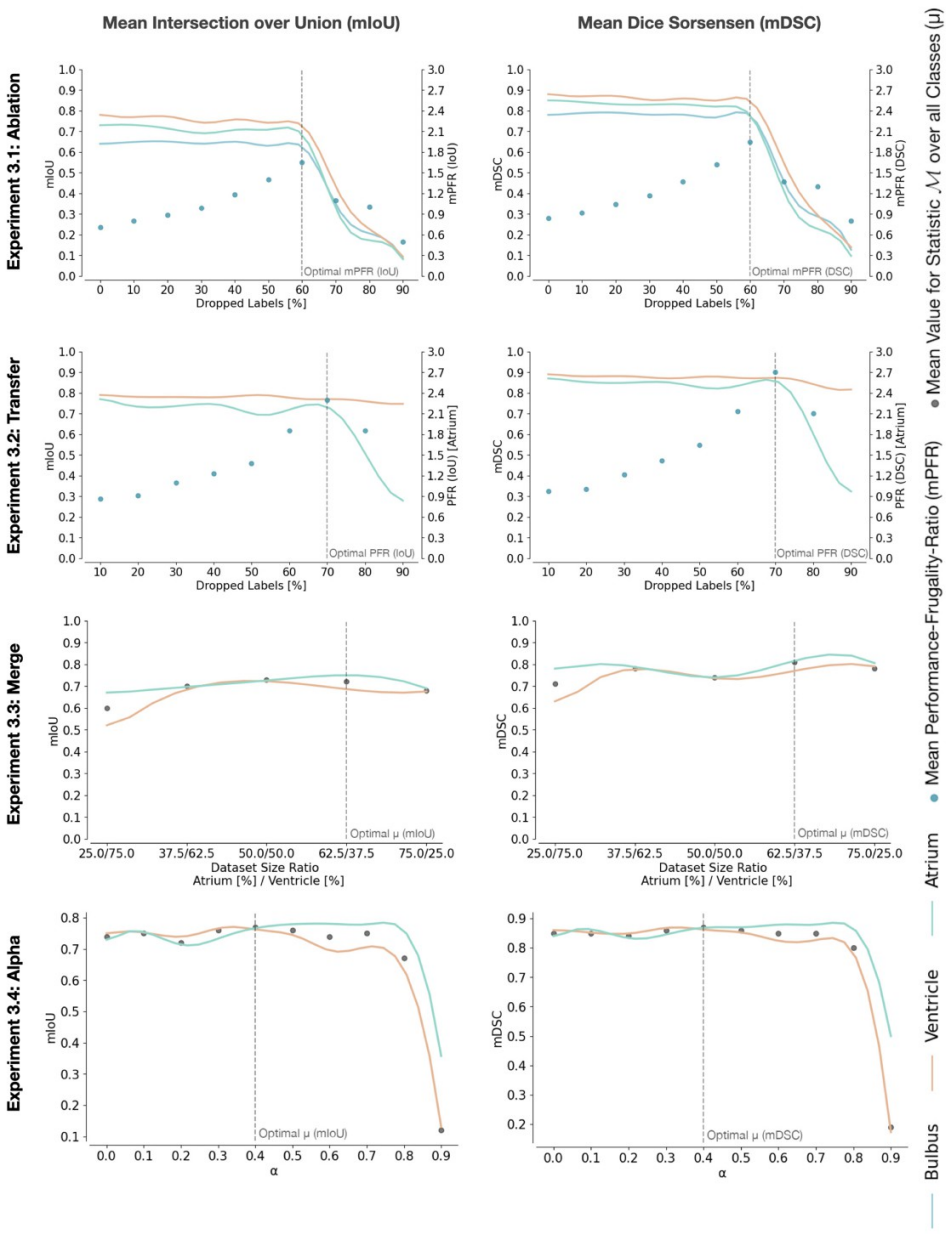
Experiments. Visualization of the conducted experiments. Each row is an experiment, each column the applied statistic. The scatter points in each plot indicate the value for the respective method to measure performance, either the mean Performance Frugality Ratio *PFR* over the observed classes or the mean value (*μ*) over the respective statistic. The optimal performance is highlighted with a vertical dotted line. The plotted values are presented as a moving average with kernel size two. The left axis corresponds to the respective statistic, the right axis, if it is present, to the respective PFR. For details see S1 File.

The first experiment focuses on the central claim of our work, demonstrating the ability to train on heterogeneously labeled data. The effect is presented on different magnitudes of heterogeneity, so labels from all classes are dropped equally with an increasing percentage. This general label ablation study does not specify a particular use-case. However, it evaluates how dropped labels influence performance, referring to the baseline of supervised training on the fully labeled dataset (dropped labels 0%). The results (see Experiment 3.1 in Fig 3) show that there is no major drop in performance up until about *ρ* = 60% dropped label share according to both the mean IoU (mIoU) and mean DSC (mDSC). Mean PFR (mPFR) also progresses alike for both metrics. After that, performance drops rapidly. At *ρ* > 70%, the network will usually converge to a state in which the background class is assumed as the correct class for all pixels. It is assumed that this behavior occurs since the background class is a composition of the other classes and hence occurs more frequently if few classes are labeled, which implicitly increases its importance while training. It is also apparent from the results that the bulbus class is the hardest to segment, which is expected since the cardiac outflow tract is not as clearly delineated as the atrium and ventricle.

### 3.2 Extending an additional class by transfer learning

For the second experiment, a situation is assumed where the dataset is fully labeled with a single class and should be extended by a second one. The samples, which are already labeled with one class, are partially extended by the labels of a second class, making the dataset heterogeneous. In contrast to the first experiment, one class remains homogeneously labeled. Here, the ventricle class is always fully labeled, and the atrium class contains a decreasing number of labels to simulate incomplete labeling processes for the atrium class. The dataset is reduced to only two classes (atrium and ventricle) for this experiment. The performance of the ventricle class stays relatively constant (which is to be expected). The atrium class shows a slowly decreasing performance when the number of dropped labels increases. Dropping *ρ* = 40% atrium class labels from our dataset results in performance deterioration of 1% in *DSC* and 2% in IoU (see Experiment 3.2 in Fig 3). If only 60% of the atrium samples are labeled, there is solely a marginal performance decrease compared to labeling all samples.

### 3.3 Merging heterogeneous labeled datasets

The third experiment simulates a situation where two datasets are sampled from the same domain but with differently labeled classes. Here, two datasets containing the labels for distinct classes should be merged without additional labeling. For this, the initial dataset is split into two distinct datasets, one having only the ventricle class labeled and the other one only the atrium class. It is essential to ensure that splits only occur at complete sequences to avoid having partial sequences in both divided datasets. Only the ventricle and atrium classes are taken into account. For evaluating the synergy effect of merging heterogeneous datasets, the dataset is split into different proportions (for example, 25% / 75% would mean that one dataset makes up 25% of the overall samples and the other one 75%). Even though the dataset sizes may differ notably in this experiment, both datasets are continuously sampled with equal amounts. To achieve equivalent sampling rates oversampling is employed, which means that samples of the minority class are randomly sampled multiple times. The results show the best performance if the two datasets are balanced (which is to be expected). This experiment shows that merging two datasets from the same domain is possible. When only one class is labeled, the evaluated models perform relatively well, even when the merged datasets are imbalanced(see Experiment 3.3 in Fig 3).

### 3.4 Trading off horizontal with vertical loss

The fourth experiment evaluates how the two individual loss functions, which make up the combined objective function used in this work, contribute to the achieved results. For the sake of comparability, the same test setup as in the experiment of Section 3.2 is used. The dataset is reduced to only the atrium and ventricle classes, and only the atrium class labels are dropped. For this experiment, the dropped label share is fixed at 50%, which means half of the atrium labels are dropped. The weighting factor *α* is modified (see Eq 5) to see how both loss functions influence the learning process. The mIoU and mDSC metrics show that performance increases with increasing weight on $L_{CAL}$, up until a limit at about *α* = 0.4, at which point the performance decreases again. Both losses are essential in this experiment since the atrium class performance increases (slightly) even after *α* = 0.4. After that, however, the performance of the ventricle class starts decreasing. The best performance can be achieved if both losses are employed at a (roughly) equal share (see Experiment 3.4 in Fig 3).

## 4 Implementation and training

The additional labels for the heartSeg dataset were annotated with the Pixel Annotation Tool (version 1.4.0) [24]. The methods presented in this work were implemented with Python (version 3.7.6), using the following packages: TensorFlow (version 2.3.0), Keras (version 2.4.3), and NumPy (version 1.18.1).

### 4.1 Data augmentation and preprocessing

Due to the small-scale nature of the presented dataset, which is typical for biomedical applications, a set of data augmentation strategies is utilized:

- Rotations in range: [0,0.3] degrees,
- Width shift in range: [-0.05,0.05],
- Height shifts in range: [-0.05,0.05],
- Shear angles in range: [0,0.05] degrees (counter-clockwise direction),
- Zoom in range: [0,0.5],
- Horizontal flips with 50% probability,
- Fill mode: Nearest.

Each sample **x** ∈ **X**_*train*_ of the training set and its corresponding ground truth label mask $\tilde{y}\in {\tilde{Y}}_{train}$ are randomly augmented within the ranges defined above. For data augmentation, Keras’ ImageDataGenerator was used. In addition to augmentation, images and their corresponding label masks were scaled to a size of 256 × 256 pixels and pixel intensities in the range of [0, 1].

### 4.2 Training

**Fig 4:**
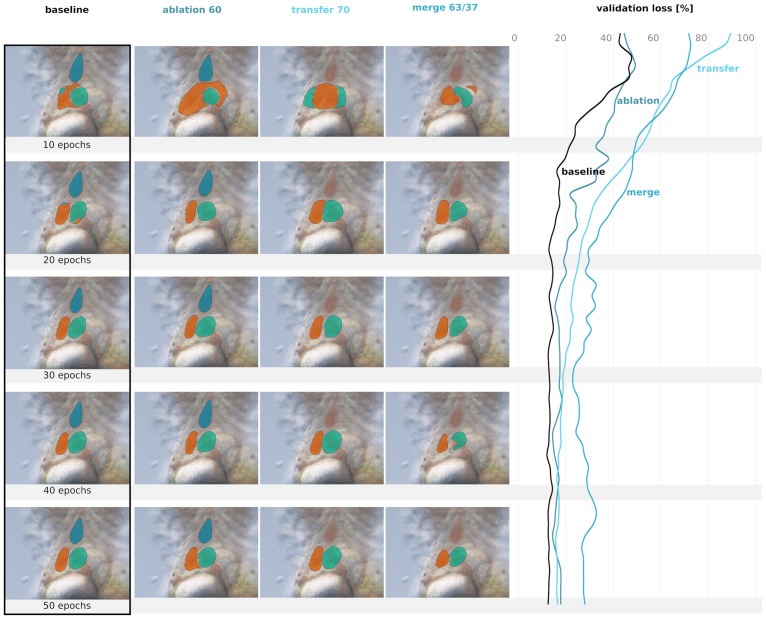
Predictions of different training schemes. For reasons of comparability, the predictions are shown for the same sample (see Fig 2). The first column presents the development of the baseline model’s prediction during the training, followed by the ablation experiment with 60% dropped labels, the transfer learning approach with 70% dropped labels, and the merge experiment with a ratio of 63% to 37%. The rows depict the predictions’ state after *e* training epochs (10, 20, 30, 40, 50). For detailed results of the experiments, see Section 3. The last column shows the validation loss development during training, presented as a moving average with kernel size three.

The U-Net model was trained until there were no improvements in the Dice loss statistic on the validation data, which happened at around 20 epochs (see Fig 4). A batch size of 11 was used, meaning there were 154 iterations, or weight updates, per epoch. For weight updates, the Adam optimizer [25] with a learning rate of 10^−3^ was used. The model was trained on an NVIDIA GeForce GTX 1080 graphics card, which requires around 160 seconds per epoch. Our U-Net implementation has 3 × 10^7^ parameters. With the early stopping approach described above, each model takes about 160 minutes to train.

### 4.3 Testing

The test data follows the dataset split of the heartSeg dataset (see Subsection 2.6). The experimental details are put forth in the results (see Section 3). Each experiment has been evaluated for ten consecutive runs. The respective relevant statistics are given for each experimental configuration with the mean and standard deviation over all runs. A comprehensive overview of the results is given in S1 File.

### 4.4 Heterogeneous label generator

Our novel heterogeneous label generator transfers a homogeneous labeled dataset into a heterogeneous one, enabling research on partially labeled datasets while retaining ground truth values for evaluation. The generator takes a dataset that contains the complete set of label masks and yields heterogeneous datasets, which only contain labels to a preconfigured degree (such as a 50% label share). Since online sampling is a requirement for many applications, the labels are dropped online while training, avoiding sample and label redundancies, keeping a persistent database. The data is always accessed on the original homogeneous dataset. This approach excludes the possibility to define a definite number of dropped labels for each class since the sample size might be unknown. Consequently, the dropped label share is defined as a relative number in the implementation.

Since any number of calls to the heterogeneous label generator should be possible without eventually encountering the dataset’s complete ground truth, the sample memory **M** is implemented. **M** stores the information on which labels have already been modified (e.g., label instance dropped) in a previous call to the function. In this way, it is ensured that an individual label $\tilde{y}$ is modified in the same way even if it is queried multiple times. After one training iteration, **M** is emptied. With that approach, the user reaches complete control over generating a heterogeneous labeled dataset without sacrificing online capabilities.

## 5 Discussion

This work provides the deep learning practitioner with a method for supervised training on heterogeneous labeled data. In particular, the method’s contribution to frugal label deployment is of interest to the biomedical research and engineering community, which often faces multiple challenges addressed in this work. Often, data acquisition is expensive, labeling is tedious and requires expert domain knowledge, and the need to fall back on multiple data sources with different label specifications is ubiquitous.

We want to draw attention to the fact that the temporal, cyclic characteristics of the extended heartSeg dataset sustain the semantic segmentation training when dropping individual labels in experiments one and two. The combined background class poses problems if only a few labels are given for a particular class. Thus, future work might advance pseudo label approaches to harness knowledge already modeled in the neural network yet missing in currently heterogeneously labeled samples.

## 6 Conclusion

The novel approaches to deploying heterogeneous labels within semantic segmentation have been presented and examined in multiple experiments, being common use-cases within biomedical semantic segmentation. As a benchmark, the multi-class segmentation dataset of Medaka’s cardiac system was developed, providing semantic segmentation label masks of three classes, together with a baseline performance (mDSC) of 84% (Table 1 in S1 File first row) by supervised training on all labels. Furthermore, a combined objective function for tasks with heterogeneous labels is introduced to cope with and utilize heterogeneous labels during neural network training, ready to be adapted to further semantic segmentation tasks and datasets. Our approaches demonstrate beneficial effects on label effort reduction while staying competitive with supervised training on the entire label set and the general cultivation of heterogeneous labeled datasets in multi-class semantic segmentation with neural networks.

The first experiment demonstrates our presented objective function’s capability to utilize heterogeneous labeled data. A common necessity in biomedical imaging tasks, where different data sources usually come with different label specifications. For 60% missing labels across all classes, the performance only deteriorates to 78% mDSC (Table 1 in S1 File seventh row), 6% below baseline performance (Table 1 in S1 File first row). The second experiment demonstrates the transfer capabilities between multiple segmentation classes within a single dataset. The presented approach is capable of extending the model by an additional class. With a reduction to merely 30% labels for one class (atrium), the approach still achieves an 84% mDSC (Table 2 in S1 File seventh row), 2% below baseline performance (Table 4 in S1 File sixth row). The third experiment demonstrates the capabilities of merging two datasets within the same domain with different classes labeled into a single training process. For balanced classes (50%/50% label share), the approach reaches a mDSC of 74% (Table 3 in S1 File third row), 12% below benchmark performance (Table 4 in S1 File sixth row). The fourth experiment demonstrates how the two individual loss functions work together to achieve the best performance. The results show that the modified Dice loss and the class-asymmetric loss play a vital role in the first three experiments. For our dataset, the best value for *α* is determined to be 0.4 (Table 6 in S1 File fifth row), though placing equal weight on each loss component is a sensible choice in practice.

This study provides approaches for the frugal labeler facing heterogeneous labeled semantic segmentation data. The heartSeg dataset, the entire training pipeline, including the heterogeneous dataset generator, and the proposed objective function’s implementation, are publicly available from the online repository (https://osf.io/uyk79/).

## Supporting information

S1 File Detailed information on the conducted experiments. Table 1: General ablation study. Table 2: Extend dataset by an additional class. Table 3: Merge two datasets. Table 4: Trading off horizontal with vertical loss.
(ZIP)
