# Supplementary material for: Methods for the frugal labeler: Multi-class semantic segmentation on heterogeneous labels
Source: PLoS One. 2022 Feb 8;17(2):e0263656. doi: 10.1371/journal.pone.0263656 (PMC8824336; doi:10.1371/journal.pone.0263656)
Supplement: S1 File — Table 1: General ablation study. Table 2: Extend dataset by an additional class. Table 3: Merge two datasets. Table 4: Trading off horizontal with vertical loss. (ZIP) [file pone.0263656.s001.zip › Supporting_Information_S1.pdf]

S1 Experiment Data

For the general ablation study, the results stay steady for up to $\rho \leq 60\%$ with a standard deviation of not more than 0.04 for the DSC metric over all classes, averaged over all ten runs. At $\rho = 60\%$, the mean DSC is 0.78 ± 0.03, which means a relative standard deviation of $\pm 3,8\%$ (see Table 1). The fully labeled ventricle class is expectedly steady in the transfer learning scenario, with only one exception at 80% dropped labels, caused by an outlier in a single experiment. At $\rho = 60\%$, the mean DSC for the atrium class is 0.81 ± 0.05, so a relative standard deviation of $\pm 6.17\%$ (see Table 2). When merging two datasets, the results for each configuration stay roughly the same over all runs, independent of how the combined dataset is split up. If the dataset is split evenly over both classes (50% / 50% share), the mean DSC is 0.74 ± 0.05, which means a relative standard deviation of $6,7\%$ (see Table 3). When trading off the horizontal with the vertical loss, the results vary very little over all runs. At $\alpha = 0.4$ the mean DSC is 0.87 ± 0.007, resulting in a relative standard deviation of just 0.8% (see Table 4).

Table 1. Evaluation results for general ablation study (dropped labels from all classes). The results are categorized according to the evaluation statistic and the amount of dropped labels. All values are averaged over ten consecutive runs, and the standard deviation is given (±). The baseline, as well as the optimal Performance Frugality Ratio PFR, is highlighted in bold.

**Dropped Labels** [%]	**mDSC** Atrium	Bulbus	Ventricle	Mean	**PFR** (Mean)
**0**	**0.85 ± 0.02**	**0.78 ± 0.02**	**0.88 ± 0.01**	**0.84 ± 0.02**	**0.84**
10	0.84 ± 0.03	0.79 ± 0.02	0.87 ± 0.02	0.83 ± 0.02	0.92
20	0.83 ± 0.01	0.79 ± 0.01	0.87 ± 0.01	0.83 ± 0.01	1.04
30	0.83 ± 0.02	0.78 ± 0.02	0.85 ± 0.02	0.82 ± 0.02	1.17
40	0.83 ± 0.02	0.78 ± 0.03	0.86 ± 0.01	0.82 ± 0.02	1.37
50	0.82 ± 0.03	0.77 ± 0.02	0.85 ± 0.01	0.81 ± 0.02	1.62
**60**	**0.75 ± 0.04**	**0.76 ± 0.03**	**0.83 ± 0.03**	**0.78 ± 0.03**	**1.95**
70	0.35 ± 0.14	0.40 ± 0.18	0.49 ± 0.20	0.41 ± 0.17	1.37
80	0.22 ± 0.30	0.28 ± 0.27	0.27 ± 0.33	0.26 ± 0.30	1.30
90	0.05 ± 0.01	0.07 ± 0.04	0.11 ± 0.07	0.08 ± 0.04	0.80

**Dropped Labels** [%]	**mIoU** Atrium	Bulbus	Ventricle	Mean	**PFR** (Mean)
**0**	**0.73 ± 0.02**	**0.64 ± 0.03**	**0.78 ± 0.02**	**0.71 ± 0.06**	**0.71**
10	0.73 ± 0.04	0.65 ± 0.03	0.77 ± 0.02	0.72 ± 0.03	0.80
20	0.71 ± 0.02	0.65 ± 0.01	0.77 ± 0.01	0.71 ± 0.01	0.89
30	0.69 ± 0.03	0.64 ± 0.02	0.74 ± 0.02	0.69 ± 0.02	0.99
40	0.71 ± 0.02	0.65 ± 0.04	0.76 ± 0.02	0.71 ± 0.03	1.18
50	0.71 ± 0.04	0.63 ± 0.02	0.74 ± 0.01	0.70 ± 0.02	1.40
**60**	**0.66 ± 0.04**	**0.61 ± 0.04**	**0.71 ± 0.05**	**0.66 ± 0.04**	**1.65**
70	0.27 ± 0.13	0.30 ± 0.15	0.38 ± 0.20	0.32 ± 0.16	1.10
80	0.17 ± 0.24	0.20 ± 0.21	0.21 ± 0.29	0.20 ± 0.25	1.00
90	0.04 ± 0.01	0.04 ± 0.02	0.06 ± 0.03	0.05 ± 0.02	0.50

Table 2. Evaluation results for the second experiment (transfer learning - extend dataset by an additional class). According to the mIoU and mDSC statistics, the results are categorized over the number of dropped atrium label masks. All values are averaged over ten consecutive runs, and the standard deviation is given (±). The baseline, as well as the optimal Performance Frugality Ratio PFR, is highlighted in bold.

**Dropped Labels** [%]	**mDSC** Ventricle	Atrium	Mean	**PFR** (Atrium)
**10**	**0.89 ± 0.02**	**0.87 ± 0.04**	**0.88 ± 0.03**	**0.97**
20	0.88 ± 0.02	0.85 ± 0.03	0.87 ± 0.03	1.00
30	0.88 ± 0.01	0.85 ± 0.07	0.87 ± 0.02	1.21
40	0.87 ± 0.03	0.85 ± 0.05	0.86 ± 0.04	1.42
50	0.88 ± 0.02	0.82 ± 0.04	0.85 ± 0.03	1.64
60	0.87 ± 0.03	0.85 ± 0.05	0.86 ± 0.04	2.13
**70**	**0.87 ± 0.02**	**0.81 ± 0.15**	**0.84 ± 0.09**	**2.70**
80	0.82 ± 0.12	0.43 ± 0.08	0.62 ± 0.10	2.10
90	0.85 ± 0.07	0.41 ± 0.06	0.63 ± 0.07	4.10

**Dropped Labels** [%]	**mIoU** Ventricle	Atrium	Mean	**PFR** (Atrium)
**10**	**0.79 ± 0.03**	**0.77 ± 0.04**	**0.77 ± 0.07**	**0.86**
20	0.78 ± 0.03	0.73 ± 0.04	0.76 ± 0.04	0.91
30	0.78 ± 0.04	0.74 ± 0.07	0.76 ± 0.06	1.10
40	0.78 ± 0.04	0.74 ± 0.04	0.76 ± 0.04	1.23
50	0.79 ± 0.04	0.69 ± 0.04	0.74 ± 0.04	1.38
60	0.77 ± 0.03	0.74 ± 0.06	0.76 ± 0.05	1.85
**70**	**0.77 ± 0.04**	**0.68 ± 0.04**	**0.73 ± 0.04**	**2.30**
80	0.75 ± 0.13	0.37 ± 0.08	0.56 ± 0.11	1.85
90	0.76 ± 0.06	0.33 ± 0.05	0.55 ± 0.06	3.30

Table 3. Evaluation results for the third experiment (transfer learning - merge two datasets). According to the evaluation statistic, the results are categorized according to the ratio between the two datasets. For example, 25.0% / 75.0% means that 25.0% of the overall samples have the atrium class labeled and 75% the ventricle class. All values are averaged over ten consecutive runs, and the standard deviation is given (±). The baseline (50% / 50% label share) and the dataset size ratio with optimal performance are highlighted in **bold**.

**Dataset Size Ratio**		**mDSC**	
Atrium [%] / Ventricle [%]	Ventricle	Atrium	Mean
25.0 / 75.0	0.63 ± 0.08	0.78 ± 0.02	0.71 ± 0.05
37.5 / 62.5	0.78 ± 0.03	0.78 ± 0.05	0.78 ± 0.04
**50.0 / 50.0**	**0.73 ± 0.05**	**0.74 ± 0.04**	**0.74 ± 0.05**
**62.5 / 37.5**	**0.78 ± 0.09**	**0.83 ± 0.03**	**0.81 ± 0.06**
75.0 / 25.0	0.78 ± 0.03	0.78 ± 0.06	0.78 ± 0.05

**Dataset Size Ratio**		**mIoU**	
Atrium [%] / Ventricle [%]	Ventricle	Atrium	Mean
25.0 / 75.0	0.52 ± 0.02	0.67 ± 0.05	0.60 ± 0.04
37.5 / 62.5	0.70 ± 0.04	0.70 ± 0.03	0.70 ± 0.04
**50.0 / 50.0**	**0.72 ± 0.08**	**0.73 ± 0.03**	**0.73 ± 0.05**
**62.5 / 37.5**	**0.68 ± 0.02**	**0.75 ± 0.04**	**0.72 ± 0.03**
75.0 / 25.0	0.68 ± 0.04	0.67 ± 0.09	0.68 ± 0.06

Table 4. Evaluation results for the fourth experiment (trading off horizontal with vertical loss). The results are categorized according to the mIoU and mDSC statistics and then over α. All values are averaged over ten consecutive runs, and the standard deviation is given ($\pm$). The baseline (only Dice loss) and the optimal performance are highlighted in bold.

$\boldsymbol{\alpha}$	**mDSC**		
	Ventricle	Atrium	Mean
**0.0**	**0.86 ± 0.023**	**0.84 ± 0.009**	**0.85 ± 0.019**
0.1	0.85 ± 0.013	0.86 ± 0.004	0.85 ± 0.010
0.2	0.85 ± 0.022	0.83 ± 0.043	0.84 ± 0.036
0.3	0.87 ± 0.014	0.85 ± 0.003	0.86 ± 0.016
**0.4**	**0.86 ± 0.000**	**0.87 ± 0.007**	**0.87 ± 0.007**
0.5	0.85 ± 0.033	0.87 ± 0.000	0.86 ± 0.027
0.6	0.82 ± 0.004	0.88 ± 0.001	0.85 ± 0.029
0.7	0.83 ± 0.005	0.88 ± 0.000	0.85 ± 0.021
0.8	0.75 ± 0.020	0.85 ± 0.029	0.80 ± 0.055
0.9	0.00 ± 0.002	0.39 ± 0.102	0.19 ± 0.205

$\boldsymbol{\alpha}$	**mIoU**		
	Ventricle	Atrium	Mean
**0.0**	**0.75 ± 0.035**	**0.73 ± 0.014**	**0.74 ± 0.029**
0.1	0.75 ± 0.020	0.75 ± 0.006	0.75 ± 0.015
0.2	0.74 ± 0.033	0.71 ± 0.063	0.72 ± 0.053
0.3	0.77 ± 0.022	0.74 ± 0.004	0.76 ± 0.025
**0.4**	**0.76 ± 0.000**	**0.77 ± 0.011**	**0.77 ± 0.011**
0.5	0.74 ± 0.050	0.78 ± 0.000	0.76 ± 0.041
0.6	0.69 ± 0.005	0.78 ± 0.002	0.74 ± 0.044
0.7	0.71 ± 0.007	0.78 ± 0.001	0.75 ± 0.032
0.8	0.60 ± 0.025	0.74 ± 0.044	0.67 ± 0.077
0.9	0.00 ± 0.001	0.24 ± 0.078	0.12 ± 0.133
